# Time-dependent histological characterization of amyloid-β induced cholinergic and glial alterations and their modulation by dehydroepiandrosterone sulfate (DHEAS)

**DOI:** 10.3389/fendo.2026.1764298

**Published:** 2026-03-23

**Authors:** Csenge Sólyomvári, Géza Makkai, Nicolas Capelo-Carrasco, Dubravka Svob Strac, Dóra Zelena, Szidónia Farkas

**Affiliations:** 1 Laboratory of Behavioral and Stress Studies, Institute of Physiology, University of Pécs, Pécs, Hungary; 2 Centre for Neuroscience, János Szentágothai Research Centre, University of Pécs, Pécs, Hungary; 3 Nano-Bio-Imaging Core Facility, University of Pécs, Pécs, Hungary; 4 Instituto de Biomedicina de Sevilla (IBiS), Hospital Universitario Virgen del Rocío/Centro Superior de Investigaciones Científicas (CSIC)/Universidad de Sevilla, Seville, Spain; 5 Centro de Investigación Biomédica en Red sobre Enfermedades Neurodegenerativas (CIBERNED), Madrid, Spain; 6 Department of Bioquímica y Biología Molecular, Facultad de Farmacia, Universidad de Sevilla, Seville, Spain; 7 Laboratory for Molecular Neuropsychiatry, Ruder Boskovic Institute, Division of Molecular Medicine, Zagreb, Croatia

**Keywords:** amyloid-β, astrocytes, cholinergic system, dehydroepiandrosterone sulfate (DHEAS), microglia, neuroinflammation, neurosteroids

## Abstract

**Introduction:**

Alzheimer’s disease (AD) is a multifactorial neurodegenerative disorder characterized by predominant - but not exclusive - pathological accumulation of amyloid-β (Aβ) in the brain. This process affects not only neurons (particularly cholinergic) but also glial cells, contributing to progressive neuronal loss and neuroinflammation. Dehydroepiandrosterone (DHEA) and dehydroepiandrosterone sulfate (DHEAS) are endogenous steroids that are hypothesized to exert neuroprotective and anti-inflammatory effects. This study aims to histologically characterize the *in vivo* temporal progression of Aβ-induced alterations in cholinergic neurons and glial morphology. Our secondary aim was to evaluate whether DHEAS protects cholinergic integrity and, if so, whether this effect is mediated through glial activation.

**Methods:**

Aβ_1–42_ was injected into the cholinergic nucleus basalis magnocellularis (NBM) region of C57BL6/J male mice and one hour later 10 mg/kg DHEAS or vehicle (0.9% saline) was applied intraperitoneally. After 3, 12 or 33 days, the mice were transcardially perfused and immunohistochemical staining was used to investigate cholinergic cell (ChAT) and fiber (AChE) loss, as well as microglia (IBA1) and astrocyte (GFAP) morphology.

**Results:**

Our findings confirmed that Aβ peptide exerted neurotoxic effects on the cholinergic system and triggered time-dependent activation in both glia cell types. Microglial cells initiated their response by day 3, adopting an amoeboid morphology, whereas delayed astrocytic reactivity was observed between days 3 and 12, demonstrated by increased ramification. DHEAS treatment preserved cholinergic fiber density, without effecting the number of cell bodies and modulated the inflammatory responses of glia cells, by decreasing the area occupied and number of microglia in a time dependent manner.

**Discussion:**

Aβ toxicity exerts time-dependent effects on both cholinergic neurons and glia cells, while DHEAS shows therapeutic promise, though its efficacy and exact mechanism require further investigation.

## 1 Introduction

Alzheimer’s Disease (AD) is the most common neurodegenerative disorder, with a complex etiology not yet fully understood. The deterioration of the cholinergic system, observed in AD, results in a significant decrease of acetylcholine (ACh). This neurotransmitter is essential for cognitive processes, such as learning and memory (1, 2). Evidence shows that the basal forebrain, especially the Meynert nucleus (in rodents the nucleus basalis magnocellularis complex, NBM) (2, 3), the brain’s main source of acetylcholine, is significantly affected (4). A central event (alongside other contributing factors) that initiates the pathogenesis of AD might be the formation and accumulation of amyloid-beta (Aβ) oligomers. This plays a crucial role in driving cholinergic dysfunction and degeneration. In the human brain Aβ accumulates in multiple phases, with cholinergic basal forebrain neurons affected from phase 3. At this stage clinical cognitive decline typically emerges, underscoring the central role of plaques in the disease progression (5). Aging appears to exacerbate Aβ toxicity, with cholinergic neurons being especially vulnerable (6). Specifically, Aβ accumulation can decrease ACh synthesis and secretion (3, 7), as well as choline uptake (8). Moreover, acetylcholinesterase (AChE), the enzyme that rapidly breaks ACh at the axon terminals (2), can interact with Aβ peptides, promoting the formation of Aβ plaques and fostering neuronal death (9). The Aβ accumulation can also influence Ca²^+^ homeostasis, leading to further cell degeneration (10). These findings suggest that injection of Aβ into NBM, which leads to cholinergic cell death, can be used as a targeted model of AD-related neuronal toxicity (11).

Neuroinflammation, attributed to activated microglia and astrocytes, is considered as a driving force in the onset and progression of AD (12, 13). Glial cells respond to Aβ plaque formation by transitioning into reactive forms (14, 15). In the early stages of the disease, reactive glia plays a beneficial role in clearing Aβ. However, prolonged activation results in chronic inflammation, characterized by elevated levels of pro-inflammatory cytokines, such as tumor necrosis factor-α (TNFα), interleukins (IL-6, IL-1β) and nitric oxide (NO), with damaging effects on the nervous system (16). Microglia activation, driven by elevated levels of Aβ and reactive oxygen species (ROS), results in impaired Aβ phagocytosis (16). In parallel with this, reactive astrocytes undergo hypertrophy and overexpress markers such as glial fibrillary acidic protein (GFAP), nestin, and vimentin (16–19). In AD, astrocytes become more sensitive to Ca²^+^ signaling, reacting more rapidly and intensely and exhibiting increased ROS production. This reduces the levels of glutathione (GSH), one of the brain’s most important antioxidants (20). Hence, mitigating inflammation could represent potential therapeutic approach to AD.

Dehydroepiandrosterone (DHEA) is an endogenous steroid that serves as a precursor for sex hormones testosterone and estrogen. It is primarily produced in adrenal glands and gonads (21, 22), but also synthesized in the central nervous system by neurons and glial cells (23, 24). DHEA is converted into its more stable, water-soluble, biologically active and major circulating form, dehydroepiandrosterone sulfate (DHEAS). Numerous studies have shown that both DHEA and DHEAS levels significantly decrease with age (25) and in AD (26–28). Our previous study showed that DHEAS mitigates Aβ-induced neurotoxicity by modulating mitochondrial function, apoptosis, and the PI3K/Akt–Bcl2 signaling pathway, and reduces Aβ plaque load in the motor cortex of 3xTg-AD mice (29). Due to its steroidal properties, it is hypothesized to exert neuroprotective and anti-inflammatory effects, which might be observed already after a single injection (24, 30, 31). Furthermore, DHEAS has been reported to modulate γ-Aminobutyric acid sub-type A receptors (GABA_A_R) and N-methyl-D-aspartate (NMDA) receptor function (32, 33), influence neurotrophic factors such as brain-derived neurotrophic factor (BDNF) and nerve growth factor (NGF) (34), and exert antioxidant effects. Moreover, DHEA increased acetylcholine (ACh) release from hippocampal neurons (35), while evidence suggests that DHEAS, rather than DHEA, more effectively enhances central cholinergic function (36). Based on this premise, these compounds emerge as promising therapeutic candidates for neurodegenerative diseases; however; so far the supporting evidence is sparse and not conclusive, and the exact mechanisms of their actions are still not clear (24, 37).

Therefore, using unilateral Aβ_1–42_ injection into the NBM as an animal model of AD, our aims were to (i) characterize Aβ_1-42_-induced alterations in cholinergic neurons and glial cells and (ii) assess the modulatory effects of DHEAS. Although Aβ_1-42_-induced neurotoxicity and inflammatory responses are well established, time-dependent alterations representing the progression of the processes are sparse, relying mostly on *in vitro* observations (38). In this study, animals were treated with water-soluble DHEAS one hour after the neurotoxic injection, and subsequent cellular and histological alterations were analyzed. Morphological examination was assessed 3, 12 and 33 days after the neurotoxic lesion, based on previous results from our laboratory demonstrating neuroprotective potential of estradiol in these timepoints (11).

## 2 Materials and methods

### 2.1 Animals

The study enrolled three-month-old male C57BL/6J mice, since a more prominent effect was expected in males due to the androgenic nature of DHEA and DHEAS (39). All animals were bred and housed at the University of Pécs, Medical School, Institute of Physiology. Mice were maintained under a 12 hours light/dark cycle (lights on at 10 a.m.) and provided food and water *ad libitum*. Mice (~25 g) were group-housed (5–6 per cage) in 325 × 170 × 140 mm cages. The temperature of the animal room was 22 ± 2 °C, and the relative humidity was 55 ± 10%. The number of animals was 6-8/group, in total 56. No mortality occurred during the study. All experiments were approved by the Workplace Animal Welfare Committee of the University of Pécs and the National Scientific Ethical Committee on Animal Experimentation of Hungary (BA02/2000-84/2022) and performed according to the European Community Council Directive recommendations for the care and use of laboratory animals (2010/63/EU). The authors complied with the ARRIVE guidelines.

### 2.2 Neurotoxic model, stereotaxic surgery

While Aβ injection into specific brain regions does not fully recapitulate all aspects of AD, compared to a genetic mouse line this neurotoxic model allows precise control over pathology and timing, allowing direct assessment of cholinergic and glial responses. In addition, the fact that NBM complex projects ipsilaterally to the somatosensory cortex (SSC) (40), enables comparison of the two hemispheres after unilateral Aβ_1–42_ injection. This eliminates interindividual differences, and reduces the number of required animals. Whereas bilateral injection is necessary for behavioral assessment (e.g., memory-related and cognitive outcomes), in our histological analysis we used unilateral administration allowing the contralateral hemisphere to serve as an internal control. This neurotoxic model also addresses the limited NBM pathology observed in transgenic models, where amyloid deposition primarily occurs in cortical, hippocampal, and amygdala regions with minimal and late basal forebrain involvement (41–43), in contrast to humans, where the Meynert nucleus is severely affected (44).

**Figure 1:**
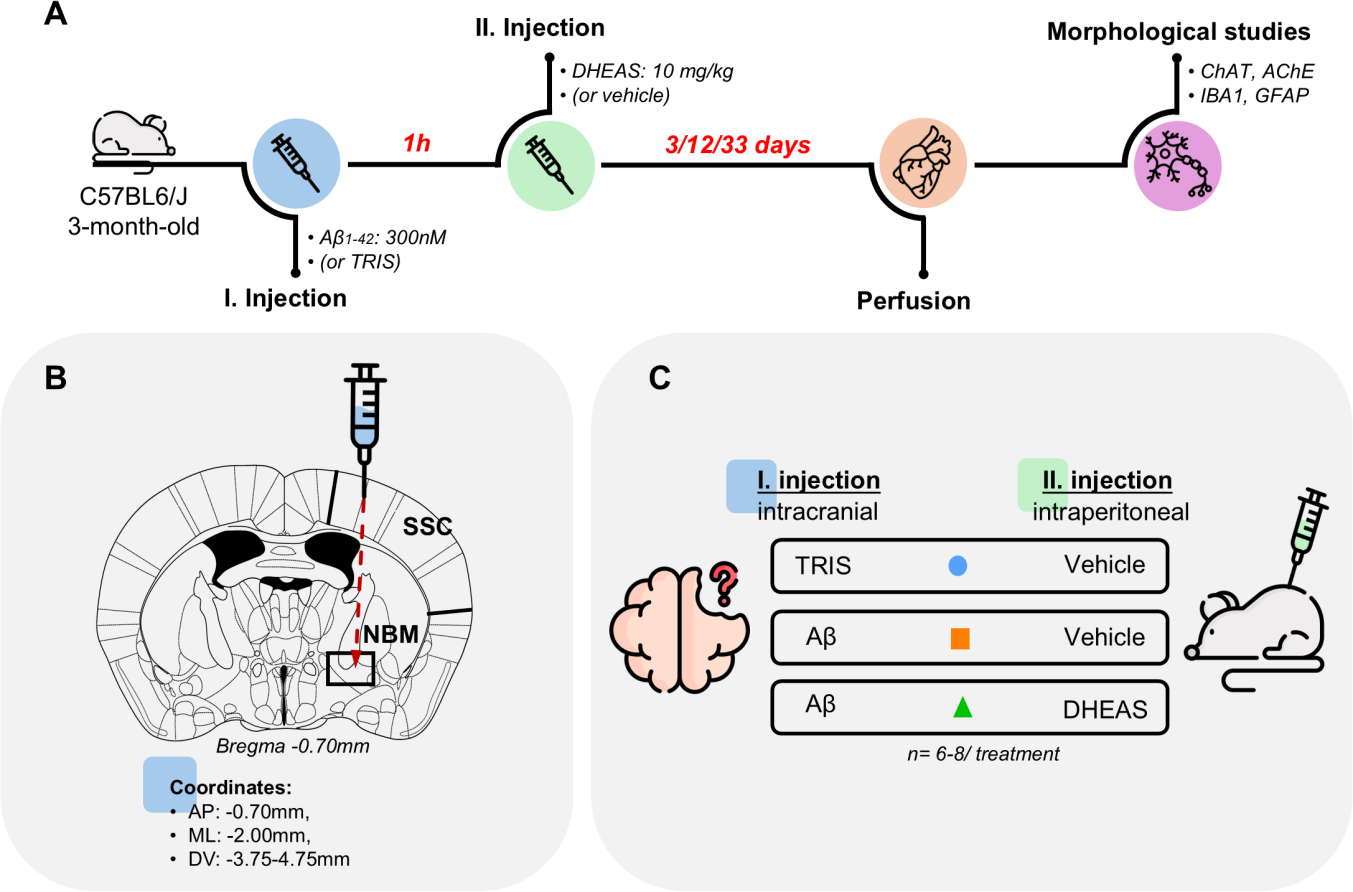
Experimental design. **(A)** The figure illustrates the chronological order of interventions, the time intervals between treatments **(red)** and the concentrations used. **(B)** Representative illustration of the stereotaxic surgery targeting the nucleus basalis magnocellularis (NBM), showing the stereotaxic coordinates and the projection area in the somatosensory cortex (SSC). **(C)** The various treatment groups. Other abbreviations: amyloid beta 1-42 (Aβ_1-42_), tris(hydroxymethyl)aminomethane buffer (TRIS), dehydroepiandrosterone sulfate (DHEAS), choline acetyltransferase (ChAT), acetylcholinesterase (AChE), ionized calcium-binding adaptor molecule 1 (IBA1), glial fibrillary acidic protein (GFAP), number of animals (n). *Icons used in***Figure 1**. Retrieved from https://www.flaticon.com/.

Aβ_1–42_ was dissolved in sterile TRIS (300 nM; #252859-500G, Merck-Sigma-Aldrich, Darmstadt, Germany, pH=7.14) and “aged” for 5 days at room temperature (RT), whereas 0.05 M sterile TRIS solution (pH=7.14) was used as a control. Aβ_1–42_ or TRIS were unilaterally stereotaxically administered to the NBM in a volume of 5×0.2 μL along the DV coordinate, under a 100 mg/kg ketamine (#A31108, Calypsol 50 mg/mL, Gedeon Richter, Budapest, Hungary) and 10 mg/kg xylazine (#QN05CM92, Sedaxylan 20 mg/mL, Dechra, Northwich, UK) anesthesia (**Figure 1B**). Unilateral injection was performed because this study focused on histological evaluation, with the contralateral hemisphere serving as a control. The stereotaxic coordinates from the bregma were: AP: -0.70 mm, ML: -2.00 mm, and DV: -3.75–4.75 mm (45). One hour after surgery, the animals received a single intraperitoneal (i.p.) injection of either vehicle (Veh, 10 mL/kg saline, 0.9% sodium chloride #07220-101-190, Molar Chemicals, Halásztelek, Hungary) or DHEAS (10 mg/kg; #SLBF3469V, Merck-Sigma-Aldrich, USA), (**Figures 1A, C**). Following three different time points (3, 12, 33 days after DHEAS treatment), the animals were anesthetized with an i.p. injection of a ketamine-xylazine cocktail (see above), and transcardially perfused with 15 mL of 0.9% sodium chloride solution, followed by 30 mL of 4% paraformaldehyde (PFA; #28794.295, VWR, Radnor, PA, USA), (**Figure 1A**). Brains were dissected and postfixed in 4% PFA for 3 hours and then dehydrated with 30% sucrose solution (#S9378-1KG, Merck-Sigma–Aldrich, Darmstadt, Germany) overnight. Coronal, 30 µm brain slices were made by a freezing microtome (SM2010 R, Leica, Chicago, IL, USA). The injection site was evaluated in all analyzed mice. Stereotaxic injections were performed using a fine glass capillary, which did not lead to visible, macroscopic changes, only to a small, cortical lesions (representative images are shown in **Supplementary Figure 1**).

### 2.3 Morphological staining

#### 2.3.1 Choline acetyltransferase

Brain slices were selected from the coordinates: bregma -0.50 mm to -0.80 mm (45). Samples were washed with 0.05M TRIS for 3x10 minutes after each step. Endogenous peroxidase was blocked by a 3% peroxide solution (H_2_O_2_, #H1009-100mL, Merck-Sigma-Aldrich, Darmstadt, Germany) for 15 minutes. A background blocking mixture (containing 0.2% Triton X-100, #20540, Renal Labor, Budapest, Hungary; 10% horse serum (HS), #H1270-100ML and 0.05M TRIS) was then applied to the samples for 2 hours. After blocking, slices were incubated for 72 hours with the primary antibody ChAT (Goat, 1:1,000; #AB144P, Millipore, Burlington, MA, USA; 0.02% TX; 5% HS in TRIS). Next, brain slices were incubated with a biotinylated secondary antibody (biotinylated anti-goat 1:200, #705-065-147, Jackson ImmunoResearch, Cambridge, UK, 0.02% TX; 5% HS in TRIS) at RT for 2 hours. An avidin–biotin kit (VECTASTAIN Elite ABC-Peroxidase Kits, PK-6100, Vector Laboratories, Newark, PA, USA) diluted in 0.02% TX; 5% HS and 0.05M TRIS was applied for 2 hours, RT. The visualization was performed by Ni-DAB (3,3’-diaminobenzidine tetrahydrochloride hydrate, #D5637-1G; nickel (II) sulfate hexahydrate, #227676-500G; D-(+)-Glucose, #G8270-1KG and ammonium chloride, #213330-500G, Merck-Sigma–Aldrich, Darmstadt, Germany) and glucose oxidase (#G7141-10KU, Merck-Sigma–Aldrich, Darmstadt, Germany). Imaging was performed using a Nikon Eclipse E1 R (Nikon, Tokyo, Japan) microscope at 4x magnification.

#### 2.3.2 Acetylcholinesterase histochemistry

To label the cholinergic fibers in the SSC an AChE histochemistry was performed (**Figure 1B**) (11). Slices were selected from the coordinates: bregma +0.10 mm to −0.70 mm (45). The first step of the staining process was a 5x1 minute sodium acetate (0.1M, pH 6; #S2889-250G, Merck-Sigma–Aldrich, Darmstadt, Germany) wash. Brain slices were then incubated 2x45 minutes in a mixture of sodium acetate buffer containing: acetylthiocholine iodide (0.05%; #A5751-1G, Merck-Sigma–Aldrich, Darmstadt, Germany), sodium citrate (0.1M; #S1804-500G, Merck-Sigma–Aldrich, Darmstadt, Germany), copper sulfate (0.03M; #209198-100G, Merck-Sigma–Aldrich, Darmstadt, Germany), and potassium ferricyanide (5mM; #244023-500G, Merck-Sigma–Aldrich, Darmstadt, Germany). The incubation was followed by a 5×1 minute sodium acetate wash. After that an ammonium sulfide (1%; #S7482142 737, Molar chemicals, Halásztelek, Hungary in sodium acetate) and then a silver nitrate mixture (1%; #S8157-10G, Merck-Sigma–Aldrich, Darmstadt, Germany, in sodium nitrate 0.1 M; #S8170-250G, Merck-Sigma–Aldrich, Darmstadt, Germany) was added. Next, the samples were washed for 5×1 minutes in sodium nitrate, followed by addition of sodium acetate. Imaging was performed using a Nikon Eclipse E1 R (Nikon, Tokyo, Japan) microscope at 10× magnification.

#### 2.3.3 Fluorescent immunohistochemistry (IBA1, GFAP)

For the examination of microglia and astrocyte morphology, the injection site was selected between bregma -0.50 mm and -0.80 mm. We analyzed the NBM and the puncture channel separately, since the latter received no or very little amount of Aβ_1–42_ but showed signs of mechanical tissue damage. During the staining, the samples were washed with 0.05M TRIS for 3x10 minutes after each step. Prior incubation of samples with primary antibodies (Ionized calcium-binding adaptor molecule 1 (IBA1), rabbit, 1:1000, #019-19741, FujiFilm Wako; GFAP: 1:1000 chicken, #NBP1-05198, Novus Biologicals), background blocking was performed (same as for ChAT staining). After 72 hour incubation with the primary antibodies, Alexa Fluor conjugated secondary antibodies (Alexa Fluor (A) A674, anti-rabbit, 1:2000, #711-605-152, Jackson ImmunoResearch; A488, anti-chicken, 1:2000, #5414658300, Eugene. OR, USA) were used. Slices were incubated with secondary antibodies for 2 h at RT. Cell nuclei were stained with Hoechst 33342 trihydrochloride trihydrate (#H3570, Invitrogen, Waltham, MA, USA). After coverslipping with ProLong Gold Antifade mounting medium (#P36934, Invitrogen, Waltham, MA, USA), imaging was performed using a Nikon Eclipse Ti2-E microscope with a Nikon C2 confocal detector (Nikon, Tokyo, Japan).

### 2.4 Image analysis

Images were analyzed using ImageJ (FIJI; National Institutes of Health, USA; version 1.54f) or CellProfiler (version 4.2.8; https://cellprofiler.org) software. All experimenters were blinded to the treatment groups.

To assess the cholinergic system, ChAT^+^ cells were counted manually on both the lesioned and non-lesioned brain hemispheres, whereas for AChE^+^ fibers the integrated optical density (IOD) was quantified between layers IV and V of the SSC, as previously described (46).

For glial reactivity, the area occupied by glial cells was measured in ImageJ. Images were acquired under identical fixed settings, regardless of animal or condition. Brightness was adjusted using the “Window/Level” function, followed by conversion to 8-bit format and color inversion. After thresholding, a binary mask was created, and the “Analyze Particles” function was applied to quantify the total area occupied by the signal of interest.

For detailed morphological analysis, CellProfiler software was used. GFAP^+^ and IBA1^+^ signals were first detected and co-localized with DAPI to confirm that the analyzed objects represented cells. A minimum overlapping threshold of 10% was applied during co-localization analysis to define positive cells, ensuring that only objects with sufficient spatial overlap between immunofluorescent markers and DAPI-stained nuclei were included in subsequent analyses. Filtered objects were skeletonized, and the following parameters were extracted:

Cell count = the total number of segmented cells identified by co-localization with DAPI.

Perimeter = the total length of the cell boundary (in pixels).

Number of branches = the number of distinct cellular processes extending from the cell body.

Number of endpoints = the number of terminal ends of cellular processes.

Due to the unilateral injection, we compared the lesioned side with the intact brain hemisphere. We calculated a ratio for all parameters except for astrocyte morphology.


Ratio=lesion side/intact side

Since reactivity on the contralateral side was low or showed no GFAP reactivity at all, normalizing to the mean of the control group would affect the time-dependent analysis, therefore for astrocyte morphology we used the raw data from the injected hemisphere.

### 2.5 Statistical analysis

Statistical analysis was performed using GraphPad Prism version 8.0.1 (GraphPad software, Inc., San Diego, CA, USA). Sample size (n=6–8 per group) was determined based on power analysis. Power analysis was performed using G*Power 3.1 for a two-way ANOVA with treatment and time as factors. Assuming α=0.05, power=0.8, 2 levels of treatment, 3 time points, and a medium effect size (f=0.50), the minimum required sample size was estimated at n=6 per group per time point. To account for potential variability, we used n=6–8 animals per group. Outliers were identified using Dixon’s Q test, and only statistically significant ones were excluded. Normality was assessed using the Shapiro–Wilk and Kolmogorov–Smirnov tests. Two-way ANOVA test (with factors treatment and time) was applied for group comparisons, and Tukey HSD test was used for *post-hoc* analysis. Pearson’s correlation was calculated between cell loss and glia markers. Data is presented as mean ± SEM. A significant difference was accepted at p<0.05.

## 3 Results

### 3.1 Morphological assessment of the cholinergic system

**Figure 2:**
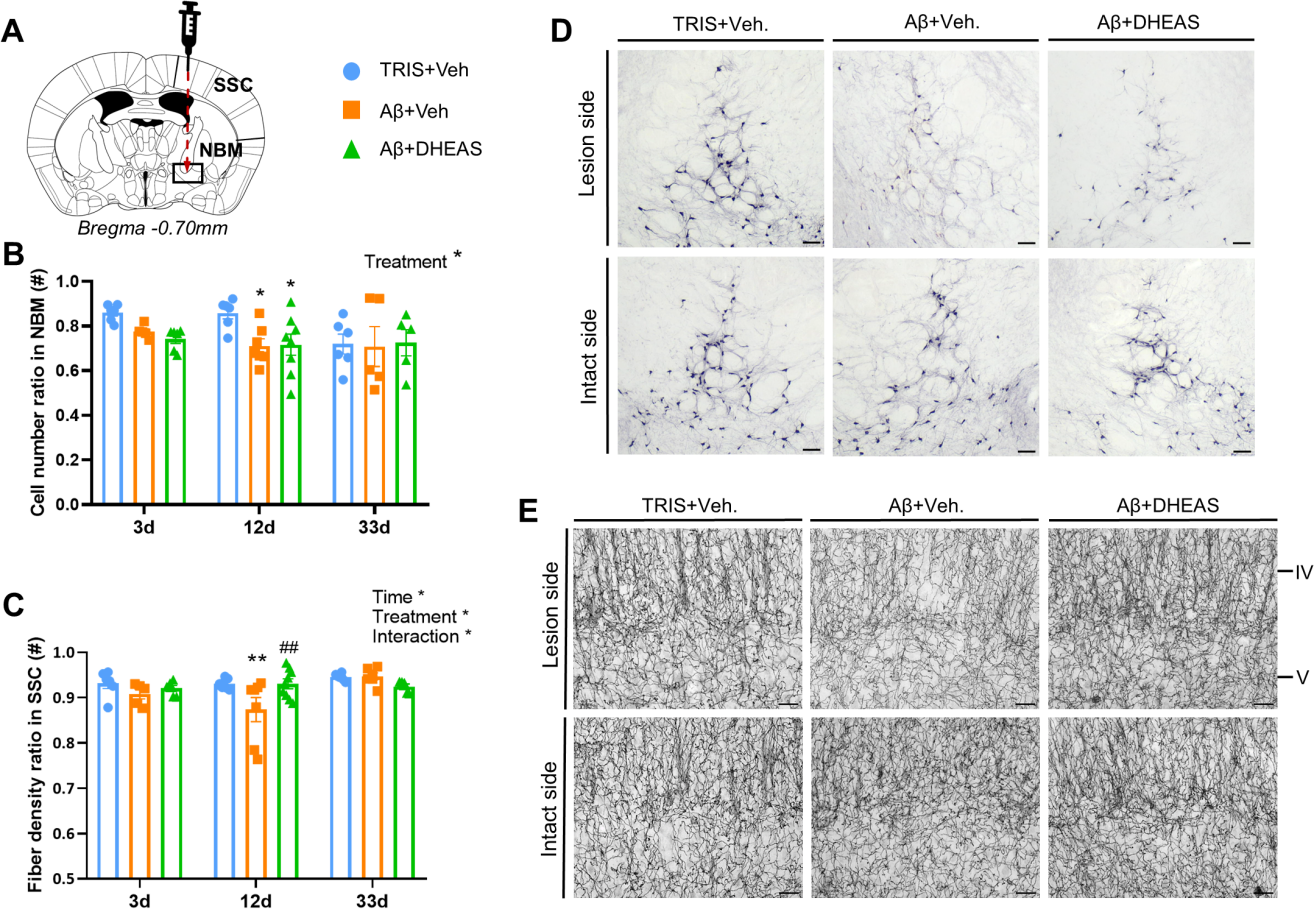
Immunohistochemical analysis of cholinergic cells and fibers. **(A)** Representative figure of the anatomical regions investigated. **(B)** Cell number changes across groups in the nucleus basalis magnocellularis (NBM). Slight decrease at 3 days post-Aβ, with significant loss by day 12 in both Aβ groups, persisting at day 33. **(C)** Changes in acetylcholinesterase (AChE) fiber density in somatosensory cortex (SSC). No change on day 3; reduced at day 12. Dehydroepiandrosterone sulfate (DHEAS) was effective and increased fiber density on day 12. On day 33, fiber degeneration showed an improving tendency, without any effect of the treatment. **(D)** Representative images of the immunohistochemical staining of choline-acetyltransferase (ChAT) positive cells on the lesioned and intact sides in the 12 days group. **(E)** Representative images of histochemical staining of AChE fibers on the lesioned and intact sides in the 12 days group. Cortical layers IV and V are visualized. Data are expressed as mean ± SEM. Differences compared to the TRIS+Veh. group are marked with asterics (*) *p<0.05, **p<0.01; while comparison to Aβ+Veh is marked with the number sign (#), ^##^p<0.01. Sample size is 6–8 animal/group. Scale bar: 20μm in D and 50μm in E.

To examine the cholinergic cell bodies, ChAT^+^ cells were counted in the NBM (**Figure 2A**). The results demonstrated significant difference between groups (treatment: F_(2,45)_=3.729, p=0.0317); but no significant time effect (F_(2,45)_=2.094, p=0.1351) or interaction (F_(4,45)_=1.037, p=0.3988), (**Figures 2B, D**). The significant Aβ_1-42_-induced cell loss was detectable only at 12 days (TRIS+Veh. vs Aβ+Veh: p=0.0398; TRIS+Veh. vs Aβ+DHEAS: p=0.0416) and cholinergic cell number remained low 33 days post-injury. However, DHEAS treatment did not exert a protective effect against cholinergic cell death at any of the examined time points.

We further investigated the density of AChE^+^ fibers in the SSC, the projection area of NBM neurons. We observed significant time (F_(2,49)_=3.748, p=0.0306) and treatment (F_(2,49)_=3.187, p=0.0500) effects, as well as their significant interaction (F_(4,49)_=2.774, p=0.0372), (**Figures 2C, E**). Specifically, although three days after the Aβ_1-42_-lesion no changes in fiber density were detected, after 12 days the Aβ_1–42_ injection significantly reduced the relative fiber density compared to control group (p=0.0034). By day 33, the Aβ_1-42_ -induced fiber density decrease showed signs of spontaneous improvement. DHEAS treatment significantly reduced the extent of AChE fiber degeneration 12 days following Aβ_1-42_-lesion (p=0.0024).

### 3.2 Glial coverage in the cortex

**Figure 3:**
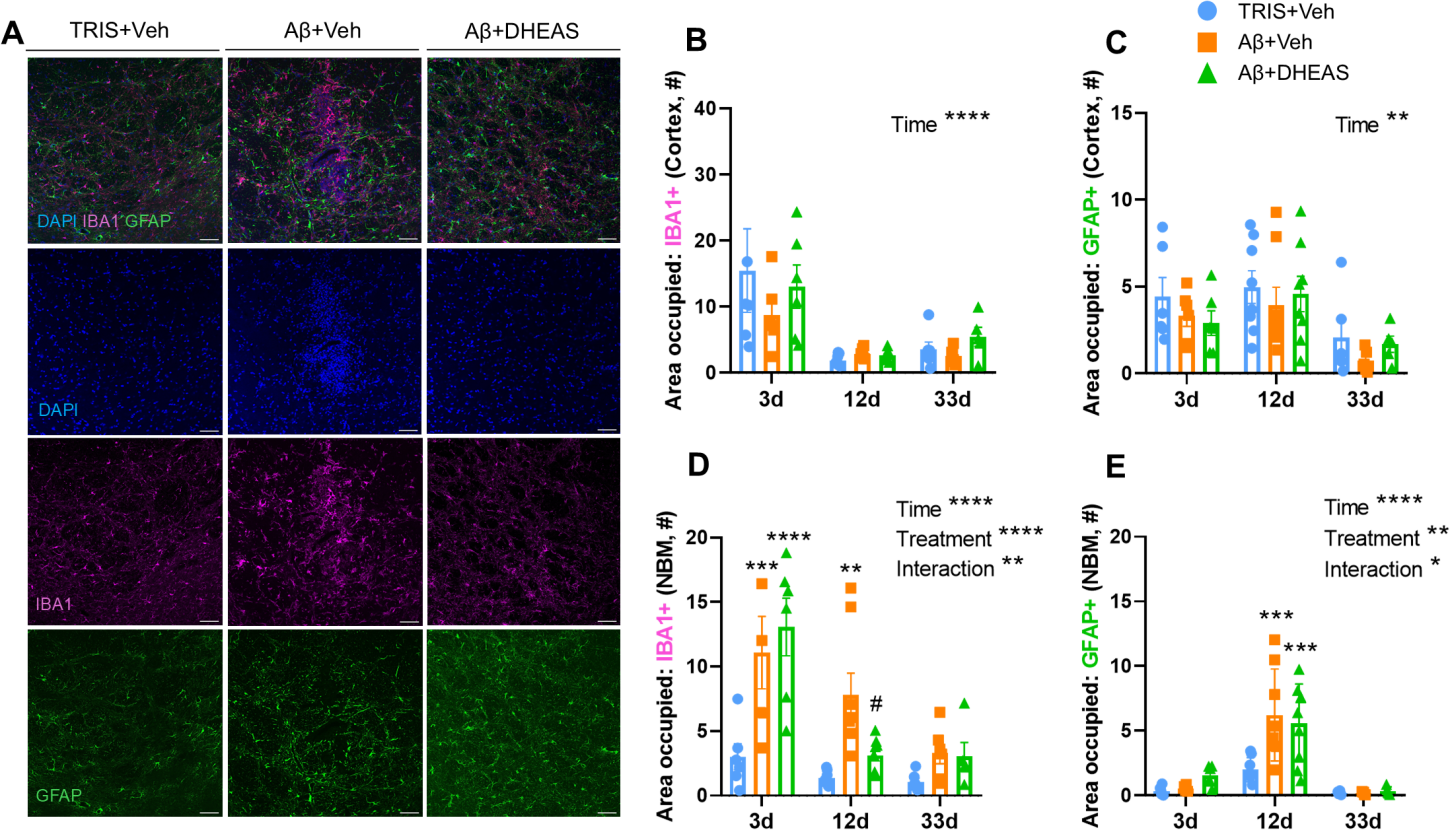
The effects of stereotaxic surgery on glia cell coverage, in the cortex and nucleus basalis magnocellularis (NBM). **(A)** Representative confocal images of the Aβ microinjection in the NBM on day 12 (labels: glial fibrillary acidic protein (GFAP), Ionized calcium-binding adaptor molecule 1 (IBA1). **(B)** Area occupied by microglia cells (IBA1) at different times after the mechanical injury in the cortex. Differences were only seen over time, with no significant variation between treatment groups. The area covered peaked on day 3 and steadily decreased to day 12 and 33. **(C)** Area occupied by astrocyte cells (GFAP) at different times. The coverage peaked on day 12 post-injection and decreased entirely by day 33. No effect of dehydroepiandrosterone sulfate (DHEAS) was detected in the cortical region. **(D)** Area occupied by microglia cells (IBA1) in the NBM. A significant increase in area coverage was observed after Aβ injection in both the 3 day and 12 days groups. However, dehydroepiandrosterone sulfate (DHEAS) reduced inflammation only at 12 days post-treatment. By day 33, we found no significant differences between the groups. **(E)** Area occupied by astrocyte cells (GFAP) in the NBM. A significant increase in astrocytic coverage was seen only 12 days post Aβ injection. By day 33, the area occupied by astrocytes decreased. No DHEAS effects were detected. Data are expressed as mean ± SEM. Differences compared to the TRIS+Veh. group are marked with asterics (*), *p<0.05; **p<0.01; ***p<0.001; ****p<0,0001. while comparison to Aβ-Veh is marked with the number sign (#) ^#^p<0.05. Sample size is 6–8 animal/group. Scale bar: 200μm.

During morphological investigation of glia cells, we first examined the injection-induced mechanical injury in the cortex. Our analysis revealed significant differences across time in both glial cell types: microglia and astrocytes (IBA1: F_(2,50)_=15.95, p<0.0001; GFAP: F_(2,49)_=7.907, p=0.0011, respectively), (**Figures 3B, C**; **Supplementary Figure 2**. for representative images). The IBA1^+^ cells demonstrated a higher area occupancy on day 3 (3 days vs. 12 days: p<0.0001; 3 days vs. 33 days: p=0.0002), which has been reduced to day 12, remaining low also on day 33. In contrast, GFAP^+^ cells showed the highest area coverage on day 12 (3 days vs. 12 days, p=0.4007), which decreased in day 33 (12 days vs. 33days, p=0.0007), (**Figure 3C**). There were no significant effects of treatment or significant interaction between treatment and time at any time point, which is in accordance with the fact that the Aβ_1-42_-lesion was not induced in the cortex (**Figures 3B, C**). Furthermore, the DHEAS treatment had no effect on mechanical injury-induced glial reactivity in the cortex.

### 3.3 Glial coverage in the NBM

In the NBM both glial cell types, microglia and astrocytes, showed significant time-related differences (IBA1: F_(2,51)_=16.87, p<0.0001; GFAP: F_(2,51)_=33.34, p<0.0001), (**Figures 3A, D, E**), mirroring the cortical pattern (**Supplementary Figure 2**. For representative images). Area covered by microglia cells demonstrated a general decline over time, being the highest on day 3 (3 days vs. 12 days, p<0.0001; 3 days vs 33 days, p<0.0001), whereas astrocyte coverage peaked on day 12 (3 days vs. 12 days, p<0.0001; 12 days vs 33 days, p<0.0001). The findings showed that in the NBM, Aβ_1–42_ treatment resulted in a time-dependent glia reactivity (IBA1: F_(2,51)_=13.84, p<0.0001; GFAP: F_(2,51)_=4.829, p=0.0120; interaction: IBA1: F_(4,51)_=3.751, p=0.0095; GFAP: F_(4, 51)_=2.971, p=0.0279) (**Figures 3D, E**). Specifically, the Aβ_1–42_ injection significantly increased the area covered by IBA1^+^ cells (microglia) on days 3 (TRIS+Veh. vs Aβ+Veh: p=0.0007), with a smaller increase on day 12 (TRIS+Veh. vs Aβ+Veh: p=0.0019) and no significant effect at day 33 (**Figure 3D**). On the other hand, DHEAS treatment significantly reduced the area covered by IBA1^+^ cells on day 12 (Aβ+Veh vs. Aβ+DHEAS: p=0.0272). In the case of astrocytes, no significant difference was observed in the covered area 3 days after Aβ_1–42_ or following DHEAS administration (**Figure 3E**). However, after 12 days, Aβ_1–42_ injection increased the area occupied by GFAP^+^ cells (TRIS+Veh. vs Aβ+Veh: p=0.0001) without significant effect of DHEAS treatment (TRIS+Veh vs. Aβ+DHEAS: p=0.0007, Aβ+Veh vs. Aβ+DHEAS: p=0.7797). By day 33 we observed a general decrease in the coverage of GFAP^+^ astrocytes in the NBM.

#### 3.3.1 Detailed morphology of the IBA1^+^ microglia

**Figure 4:**
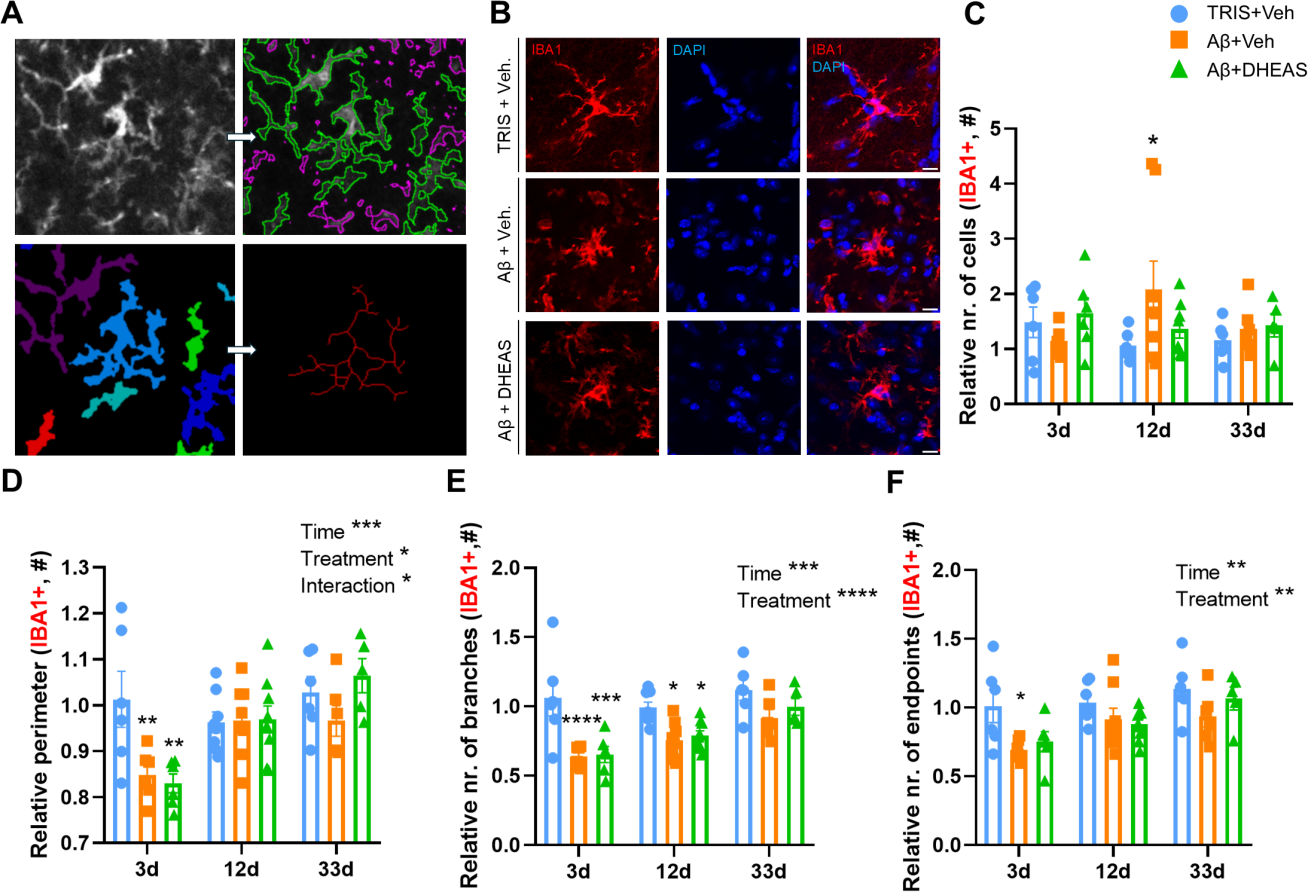
Microglia (IBA1) cell morphology in the nucleus basalis magnocellularis (NBM): **(A)** Representative figure showing detailed morphological analysis using CellProfiler software. The pictures illustrate the identification of microglia cells using DAPI and Ionized calcium-binding adaptor molecule 1 (IBA1) staining and shows the skeletonization and endpoint definition. **(B)** Representative confocal images of microglia cells ramification in the 3 days group. These images clearly showed a reduction in cell ramification and a transition to amoeboid morphology following the Aβ injection. **(C)** Number of IBA1^+^ cells at different time points. A significant increase can be seen 12 days after the injection in the Aβ+vehicle treated group. **(D)** Perimeter at different times. The perimeter significantly decreased 3 days after Aβ injection compared to the control group. Perimeter shows a sign of improvement over time. **(E)** Number of branches at different time points. The branch number significantly decreased 3 and 12 days after Aβ injection compared to the control group. **(F)** Number of endpoints at different times. This parameter significantly decreased 3 days after injection compared to the control group. Dehydroepiandrosterone sulfate (DHEAS) and Aβ groups showed similar amoeboid-like morphology, but Iba^+^ cell numbers resembled controls. Data are expressed as mean± SEM *p<0.05; **p<0.01; ***p<0.001; ****p<0,0001. Sample size is 6–8 animal/group. Scale bar: 10µm.

At the site of injection (i.e., in the NBM) Aβ_1–42_ administration increased the relative IBA1^+^ cell number at 12 days (TRIS+Veh. vs Aβ+Veh: p=0.0254), (**Figure 4C**), which was not detected in any other group (i.e., 3 or 33 days and at 12 days: TRIS+Veh. vs Aβ+DHEAS treated: p=0.6949). All other investigated morphological parameters of the IBA1^+^ microglia showed time-dependent changes (relative perimeter: F_(2,51)_=9.035, p=0.0004; interaction: F_(4,51)_=3.594, p=0.0117; relative number of branches: F_(4,51)_=9.403, p=0.0003; relative number of branch endpoints: F_(2,50)_=6.392, p=0.0034; **Supplementary Figure 2**. for representative images), with no differences observed on day 33 after Aβ_1–42_ injection. On day 3, treatment, more specifically the Aβ_1–42_ injection reduced the perimeter of the microglia cells (F_(2,51)_=3.758, p=0.0300; TRIS+Veh. vs Aβ+Veh: p=0.0044), the number of branches (F_(2,51)_=18.47, p<0.0001; TRIS+Veh. vs Aβ+Veh: p<0.0001), or the number of branch endpoints (F_(2,50)_=6.794, p=0.0025; TRIS+Veh. vs Aβ+Veh: p=0.0131). However, DHEAS did not exert any significant effects on microglia perimeter (TRIS+Veh vs. Aβ+DHEAS: p=0.0014), (**Figure 4D**), the number of branches (TRIS+Veh vs. Aβ+DHEAS: p=0.0001), (**Figure 4E**), or the number of branch endpoints (TRIS+Veh vs. Aβ+DHEAS: p= 0.8329), (**Figure 4F**) on day 3. Similar alteration, albeit with a lower magnitude, was detected on day 12 (TRIS+Veh. vs Aβ+Veh: p=0.0126; TRIS+Veh vs. Aβ+DHEAS: p= 0.0324).

#### 3.3.2 Detailed morphology of the GFAP+ astrocytes

**Figure 5:**
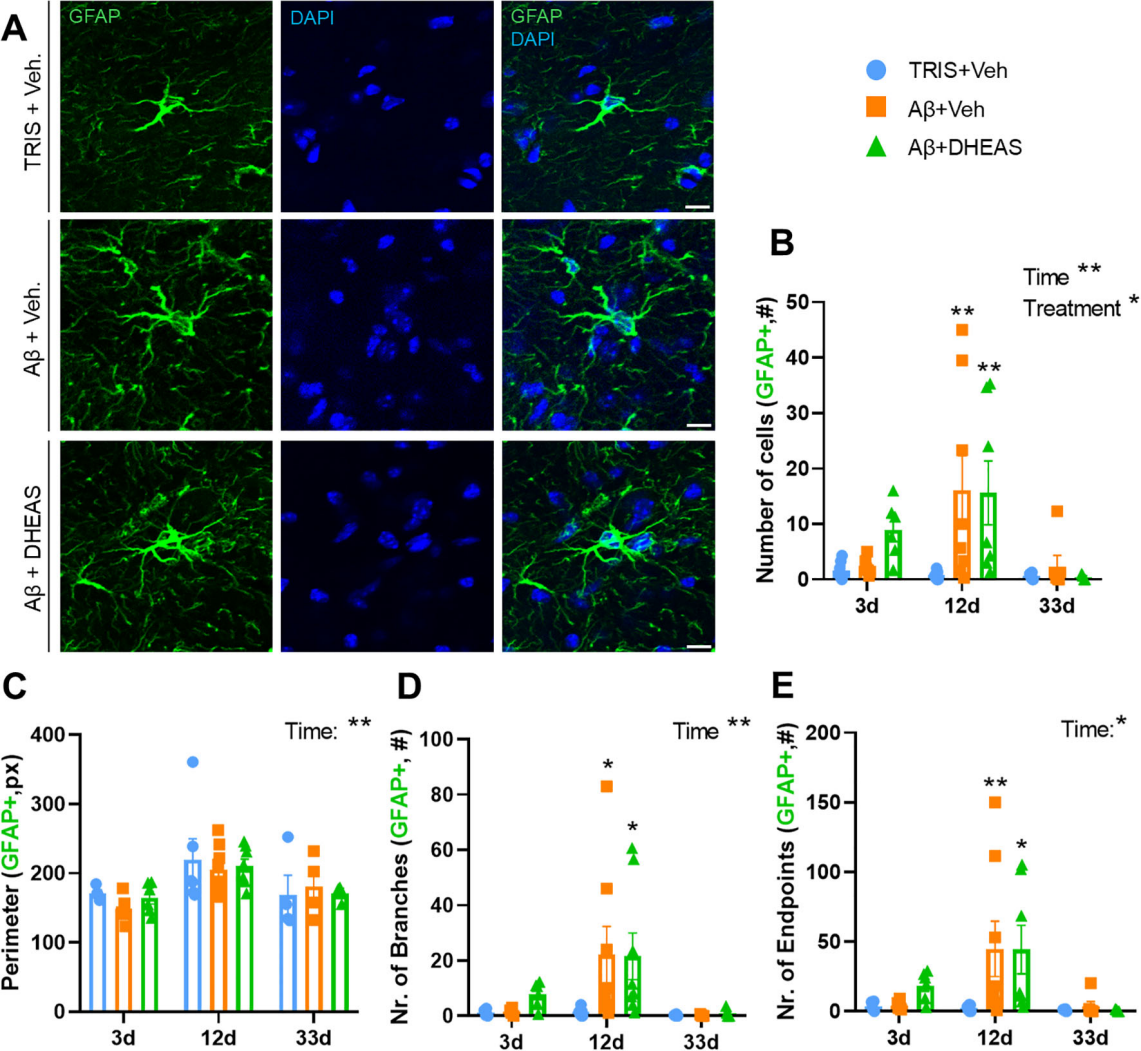
Astrocyte (GFAP) cell morphology in the nucleus basalis magnocellularis (NBM): **(A)** Representative confocal images of astrocyte cell anatomy. Increased ramification was detected after both Aβ + Vehicle and Aβ + Dehydroepiandrosterone sulfate (DHEAS) treated groups compared to control. **(B)** Number of glial fibrillary acidic protein (GFAP) positive cells at different time points. Amyloid injection significantly increased the number of cells on a day 12, which was reduced on day 33. **(C)** Perimeter at different times. This parameter showed only time-dependent changes, with the highest values on day 12. **(D)** Number of branches at different time points. The branch number significantly increased 12 days after injection compared to the control group. **(E)** Number of endpoints at the different time points. Number of endpoints significantly increased 12 days after injection. No DHEAS effect was detected, since this group showed the same morphology as the Aβ + Vehicle treated animals. Data are expressed as mean± SEM *p<0.05; **p<0.01. Sample size is 6–8 animal/group. Scale bar: 10µm.

In case of the astrocytes, all examined morphological parameters in the NBM showed time-dependent changes (cell number: F_(2,48)_=5.867, p=0.0053; perimeter: F_(2,39)_=8.001, p=0.0012; relative number of branches: F_(2,49)_=6.043, p=0.0045; relative number of branch endpoints: F_(2,48)_=5.935, p=0.0050), (**Figures 5B–E**; **Supplementary Figure 2**. for representative images), with the highest values on day 12 post-injection. Treatment, more specifically Aβ_1–42_ injection significantly increased the number of cells (F_(2,48)_=3.484, p=0.0387; TRIS+Veh. vs Aβ+Veh: p=0.0037), (**Figure 5B**), the number of branches (TRIS+Veh. vs Aβ+Veh: p=0.0141), (**Figure 5D**) and the number of branch endpoints (TRIS+Veh. vs Aβ+Veh: p=0.0090), (**Figure 5E**) on day 12, without significant effects of DHEAS on the number of cells (TRIS+Veh vs. Aβ+DHEAS: p=0.0069), (**Figure 5B**), the number of branches (TRIS+Veh vs. Aβ+DHEAS: p=0.0175), (**Figure 5D**), or the number of branch endpoints (TRIS+Veh vs. Aβ+DHEAS: p=0.0128), (**Figure 5E**). On the other hand, analysis of the relative perimeter revealed no significant differences between the groups.

### 3.4 Correlation between cholinergic cell loss and glia markers

At day 3, the correlations between cholinergic cell loss in the NBM and certain microglial parameters (specifically the number of branches and endpoints, both p<0.01) were positive, whereas the correlations with astroglial parameters (all studied, p ≤ 0.01) were negative (**Supplementary Table 1**). At day 12, no significant correlation was found between cholinergic cell loss in the NBM and any of the glial parameters examined. By day 33, the GFAP-based parameters were positively correlated with cell loss (all parameters, at least p<0.05; **Supplementary Table 1**).

## 4 Discussion

Our findings indicated that the Aβ_1–42_ peptide has time-dependent neurotoxic effects on the cholinergic system and the triggered inflammatory response can be one of the contributing factors. Damage to cholinergic neurons became particularly pronounced on day 12, whereas inflammatory responses followed distinct patterns in microglial and astrocyte cells. Microglia activation peaked on day 3 and was gradually reduced afterwards, whereas astrocyte activation was delayed, reaching a peak on day 12. Based on correlations, microglia appear to act as an aggravating factor during the early stage, whereas astrocytes seem to be protective. At later stages, however, astroglia appear to become the detrimental factor. In addition, we confirmed protective DHEAS effects on cholinergic fibers, however its anti-inflammatory properties limited to the area occupied (**Figure 3D**) and number (**Figure 4C**) of microglial cells. Thus, anti-inflammatory effects of DHEAS are likely minor, need a prolonged time (not observable at day 3), (**Figure 3D**) and do not influence glial reactivity caused by mechanical injury (**Figure 3B**).

Aβ_1–42_ injection into the NBM is a widely used method for modeling neurotoxicity in AD (47). We confirmed the induced cholinergic cell loss and reduced the fiber density in the ipsilateral projection area (SSC), which effect peaked on day 12 post-injection (11). The loss of group differences observed at day 33 may reflect the activation of compensatory mechanisms, e.g. increased AChE activity (48). Several studies have reported up to a 55% reduction in cortical cholinergic fiber density in AD patients (49). Other reports described a marked decrease in cholinergic arborization, particularly in the cortex and hippocampus, which was closely associated with inflammation (50). Thus, the effects of DHEAS on cholinergic fiber density may be critical to AD pathomechanisms, particularly in relation to its anti-inflammatory properties. Our experiment was based on a previous study using a similar protocol with 17β-estradiol (E2) administration, which also led to similar results (i.e., no effect on cholinergic cells and an increase in fiber density in the SSC (11)). We may hypothesize that DHEAS and E2 may act through similar pathways, as DHEAS is a precursor of both estrogen and androgen. However, DHEAS also has a direct, but non-specific affinity for steroid receptors (51–53). In addition, DHEAS likely exerts its effects through non-genomic targets (54). Furthermore, it exhibits direct neuromodulatory effects on both inhibitory (GABAergic) and excitatory (glutamatergic) neurotransmission. Specifically, DHEAS is an allosteric antagonist of GABA_A_R receptors (32, 55) and acts as a positive modulator of NMDA receptors (33, 56–58), with specific binding sites (59). The protective effect of DHEAS on fiber density might also be due to its ability to promote the production of NGF and BDNF (34, 60), both directly and via estrogen receptor-mediated pathways (61). Axons and dendrites possess local translation machinery responsive to neurotrophic factors, and BDNF is well known to promote dendritic spine formation, synaptic maintenance, and axonal integrity. Furthermore, DHEAS was recently discovered to maintain synaptic homeostasis and increase spine density (62). In addition, DHEAS can bind to plasma membrane receptors, such as tropomyosin kinase receptor (Trk)-A and p75 neurotrophin receptor (p75NTR), with important roles in proliferation and neuroprotection (31). Moreover, DHEAS may influence microtubule-associated proteins (MAPs), stabilizing microtubules in axons, as evidenced by its binding to the dendritic MAP2C (63). Taken together, these molecular mechanisms may underlie the protective effects of DHEAS on arborization in the cholinergic system. The findings suggest that DHEAS acts locally in axons and dendrites, promoting cytoskeletal stability, enhancing local trophic signaling, scavenging reactive oxygen species, and reducing excitotoxicity. However, it may fail to rescue the soma if nuclear stress pathways, mitochondrial damage, or severe pathology are already present.

The impact of inflammation on the central nervous system depends on its intensity and duration. While brief, controlled responses are protective, chronic or excessive inflammation may become harmful (64–66). Prolonged exposure to pro-inflammatory molecules further aggravates neurodegeneration (66–68). However, our results indicate a short and controlled inflammatory response, as by day 33 most analyzed parameters had returned to baseline or were similar to control group. This data suggests a potential limitation of this model. Specifically, while chronic, uncontrolled inflammation is a hallmark of AD, the neurotoxic injection in our study induced only a transient inflammatory response. Nevertheless, our findings highlighted the crucial role of glial cells in regulation of the inflammatory response.

The microinjection procedure itself caused mechanical injury, triggering microglial and astrocyte reactivity along the needle tract in the cortex. As no substance was deposited in this region, the observed effects resulted solely from physical trauma. It is well known that traumatic brain injury induces neuronal death and axonal degeneration (69). This is consistent with our findings obtained on day 33 in control animals (injected with TRIS), demonstrating reduced number of ChAT^+^ cells compared to the control, non-injected site (**Figure 2B**). The two types of glial cells were activated by the trauma at different times, in line with their distinct functions. Previous studies have shown that microglia activation may precede and influence astrocyte reactivity (70), potentially through the release of cytokines, which in turn enhances astrocytic response (24). Indeed, a recent study has demonstrated that in absence of any external stimuli, microglial activation induces a widespread astrocyte reactivity which is removed after depleting microglia by PLX3997 (71). After the injury, microglia cells immediately migrate to the site of damage to eliminate toxic substances, and this early inflammatory response subsides over time. This data is in line with our results on microglia, which showed increased occupied area and amoeboid morphology already on day 3. In contrast, astrocyte activation begins later and leads to glial scar formation, due to an attempt to restore tissue integrity (72), which we observed on day 12.

In the NBM microglia cells also exhibited earlier reactivity compared to astrocytes (**Figures 3D, E**). The characterization of microglial morphology in various diseases remains an active area of research (15, 73, 74). Microglia plasticity was already observed by Rıo-Hortega in 1919 (summarized by: (75)) suggesting its two main states: a „resting” state, characterized by highly ramified morphology with limited phagocytic and migratory activity; and an „active” state. The activated microglia adopt an amoeboid shape with increased motility, phagocytic capacity, and proliferative abilities (15, 73, 74). Nonetheless, this duality is being reconsidered in the field, in an attempt of defining the real status of microglia (76). Thus, we analyzed multiple parameters rather than relying on a dual scoring system. Three days after Aβ_1–42_ injection, microglia displayed an amoeboid morphology supported by the increased area occupied, reduced perimeter and number and endpoints of branches (**Figure 4**). Based upon the positive microglia-cholinergic cell loss correlation at D3 this activated microglia might have contributed to the cholinergic cell loss at D12, which might reflect that transient inflammatory signaling - likely initiated by cytokine and chemokine production - precedes structural damage. Indeed, microglia depletion prevented trauma-induced cognitive decline (77), supporting the damaging role of the microglia at early stages. Interestingly, by day 12, the number of microglia cells increased in the Aβ_1–42_ injected NBM (72), which may suggest a prolonged or secondary inflammatory response to the toxic substance. This may be explained by the fact that Aβ toxicity was insufficient to induce microglial death, allowing surviving cells to remain active and proliferate through IL-1β–mediated trophic signaling (78).

Similar to microglia, the reactivity states and molecular profiles of astrocyte cells also exhibit a transition, depending on the extent of damage and inflammation (17, 79, 80). During reactivity, they show hypertrophy, with an increase in both their size and the number of branching in their processes (17–19). In the resting state, an astrocyte has fewer processes and shows low GFAP expression (79, 81). Additionally, non-reactive astrocytes are spaced farther apart with minimal overlap (17). In the case of severe injury, neurodegenerative effects, or chronic inflammation, reactive astrogliosis is characterized by a significant increase in gene expression and cellular hypertrophy (17, 80). The data obtained in our study is consistent with the literature as Aβ_1–42_ injection increased the number of astrocytes, branches, and endpoints (**Figure 5**), indicating a more ramified morphology 12 days post-injection. Although the observed morphological alterations suggest increased glial reactivity, future studies incorporating mRNA and protein markers (e.g., *Il1b*, *Tnfa*, *Trem2*, *Clec7a*, *Cd45*) are warranted to provide a more comprehensive characterization of glial activation beyond morphology. Interestingly, the early negative and later positive correlations between astrocyte markers and cholinergic cell loss suggest a shift in astrocyte function from an initially protective role to a later detrimental one. Several studies support the protective role of astrocytes (82), which may subsequently transition to a harmful phenotype (83), presumably due to activation by microglia (84, 85). Thus, the observed changes might represent apadtive, rather than compensatory responses.

DHEAS treated group often displayed outcomes indistinguishable from the Aβ+Veh. group, whereas mild anti-inflammatory effects of DHEAS were primarily observed on day 12. Previous findings suggested that DHEAS anti-inflammatory effects (24) could be exerted via binding to TrkA receptors and activation of protein kinase B/cAMP response element-binding protein (AKT/CREB) pathway. This indirectly inhibits the expression of inflammatory genes and reduces secretion of pro-inflammatory cytokines from microglia (30). Furthermore, DHEA has been shown to induce JMJD3 (an inflammation-sensitive histone demethylase) expression and promote an anti-inflammatory microglial phenotype in hemoglobin-induced neuroinflammation (86). Through its action on oligodendroglia cells, DHEAS may reduce demyelination and axon loss. Moreover, DHEAS was also shown to decrease IL-1β and interferon-gamma (IFNγ) levels in the spinal cord (87). After being transformed into androstenediol, DHEAS can modulate estrogen receptor β, thereby suppressing the inflammatory response of glia cells (88). It has been reported to exert antioxidant effects in microglia, as it reduces LPS-induced nitrite production and iNOS protein expression, suggesting a post-transcriptional mechanism that limits reactive nitrogen species generation (89). Chronic DHEA treatment - similar to a single DHEA dose - reduced cocaine-induced astroglia volume in the dentate gyrus of rats (90). Another possible molecular effect of DHEA is the enhancement of glutamate uptake by astrocytes, by promoting the insertion of the glutamate transporter (GLT-1) into the cell membrane (91). Previous studies have shown that DHEAS can reduce reactive astrocytes, as indicated by decreased S100β-positive cells in a cocaine self-administration model (90) and attenuate astroglial activation in an iron-induced post-traumatic epilepsy model (92). In our study, the lack of detectable GFAP morphological changes may reflect the treatment window used. Moreover, functional or biochemical changes, such as cytokine secretion, may occur without detectable morphological alterations (79, 93). Future studies using chronic or later administration, as well as complementary functional or molecular markers (e.g., S100β or ALDH1L1), could provide additional insight into astrocytic modulation by DHEAS. Furthermore, DHEAS may shift the excitatory/inhibitory balance, indirectly influencing microglial activation and astrocytic metabolic and inflammatory responses, as both glial cell types are highly sensitive to neuronal activity patterns (94, 95). This mechanism may counteract the anti-inflammatory effect of DHEA masking any changes. DHEA also appears to reduce astroglia activation following denervation, although this may partly be due to its conversion into estrogen (96). All in all, previous findings suggest that glial cells may play a central role in mediating the effects of DHEAS. The limited effectiveness of DHEAS administration in our study might be due to the timing or characteristics of the treatment.

Since, besides Aβ, tau hyperphosphorylation also plays a central role in the pathomechanism of AD, it is important to assess our results in the context of tau pathology. Tau accumulation in basal forebrain cholinergic neurons inhibits their firing, and number, and extracellular tau may increase this loss via interactions with M1/M3 muscarinic receptors (97–99). Furthermore, transgenic mouse models expressing various pathological pTau mutations exhibited a reduction in ChAT-positive basal forebrain neurons, concomitant with impairments in spatial learning and enhanced microglial activation (50). Notably, microglia-driven inflammation not only responds to tau pathology but also actively promotes it, driving the progression of tauopathies and facilitating the spreading of pathological tau throughout the brain (100, 101). Additionally, tau might induce proinflammatory astrocytic activation (102), which might also contribute to neurodegeneration (103). In relation to DHEA, its *in vitro* administration elevated the level of Tau, but not its phosphorylation (104), questioning their interaction.

In summary, Aβ_1–42_ administration demonstrated a neurotoxic effect, as evidenced by the reduced number of ChAT^+^ cells in the NBM and their decreased projections in the SSC. The time-dependent microglial cell activation in the NBM may precede, and aggravate the process. DHEAS protective actions against Aβ-neurotoxicity were observed on day 12 on the cholinergic fibers, with mild anti-inflammatory effects on glial cells. Our findings primarily contribute to the better understanding of complex AD pathogenesis in a histological level, while also suggesting therapeutic potential of DHEAS. However, additional studies are required to substantiate this possibility.

## Data Availability

The raw data supporting the conclusions of this article will be made available by the authors, without undue reservation.
