# Supplementary material for: Time-dependent histological characterization of amyloid-β induced cholinergic and glial alterations and their modulation by dehydroepiandrosterone sulfate (DHEAS)
Source: Front Endocrinol (Lausanne). 2026 Mar 23;17:1764298. doi: 10.3389/fendo.2026.1764298 (PMC13050736; doi:10.3389/fendo.2026.1764298)
Supplement: Supplementary file 1 [file Supplementaryfile1.docx]

Supplementary Material

Time-dependent histological characterization of amyloid-β induced cholinergic and glial alterations and their modulation by dehydroepiandrosterone sulfate

Csenge Sólyomvári ^1,2^, Géza Makkai ^3^, Nicolas Capelo-Carrasco ^4,5,6^, Dubravka Svob Strac ^7^, Dóra Zelena ^1,2*†^, Szidónia Farkas ^1,2†^

^1^Laboratory of Behavioral and Stress Studies, Institute of Physiology, University of Pécs, Pécs, Hungary

^2^Centre for Neuroscience, János Szentágothai Research Centre, University of Pécs, Pécs, Hungary

^3^ Nano-Bio-Imaging Core Facility, University of Pécs, Pécs, Hungary

^4^Instituto de Biomedicina de Sevilla (IBiS), Hospital Universitario Virgen del Rocío/CSIC/Universidad de Sevilla, Manuel Siurot s/n, 41013, Seville, Spain

^5^Centro de Investigación Biomédica en Red sobre Enfermedades Neurodegenerativas (CIBERNED), Valderrebollo, Madrid, Spain.

^6^Department of Bioquímica y Biología Molecular, Facultad de Farmacia, Universidad de Sevilla, Tramontana, Seville, Spain

^7^Laboratory for Molecular Neuropsychiatry, Ruder Boskovic Institute, Division of Molecular Medicine, Zagreb, Croatia

**^†^**Equal contribution


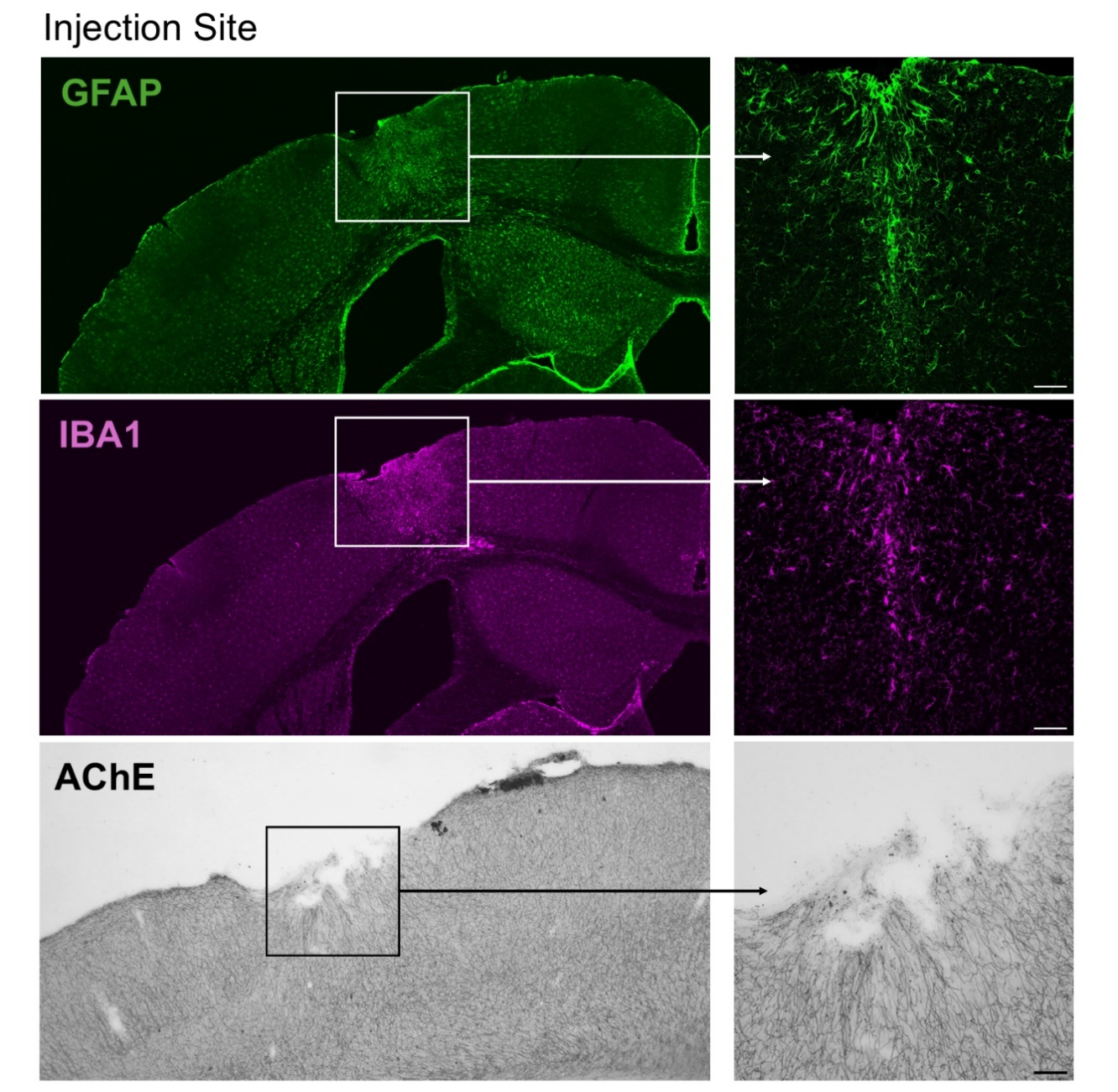


**Supplementary Figure 1. Mechanical injury in the cortex.** The injection itself can cause mechanical injury0. which was evaluated in all assessed mice. The injection site showed increased GFAP and IBA1 immunoreactivity in the cortex0. along with mild morphological alterations in all animals. The area occupied by GFAP and IBA1 positive cells was quantified and is shown in Figure 3B and C. AChE staining was performed anterior to this region0. where the injection tract was no longer detectable and where NBM neurons project. As the procedure involved stereotaxic injection using a fine glass capillary0. no macroscopic alterations were observed along the injection tract or within the NBM. The NBM was systematically analysed in all assessed mice0. and the results are presented in Figures 2–5. During analysis a ratio was calculated between the lesion and non-lesion site. Scale bar on inserts: GFAP and IBA1: 200 μm; AChE: 20μm.


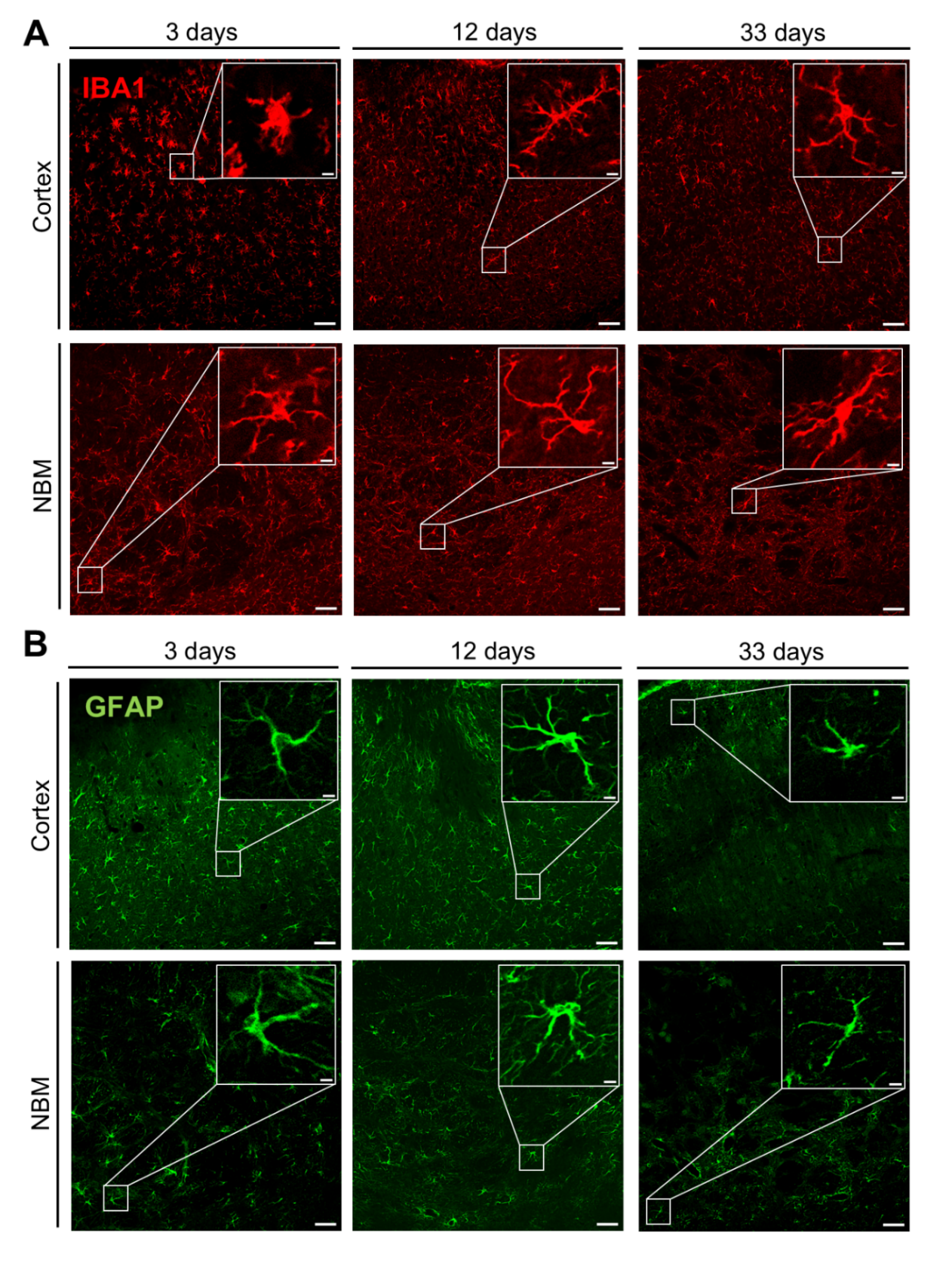


**Supplementary Figure 2. Temporal and morphological changes in microglial and astrocytic reactivity.** (A) IBA1 positive microglial morphology at different time points. An increased area occupied0. and amoeboid-like morphology were detected on day 3 in both cortical and basal forebrain (NBM) regions; quantification is shown in Figure 3. (B) GFAP-positive astrocytes showed an increased occupied area on day 120. along with enhanced ramification; quantification is shown in Figure 3. Scale bars: 50 µm for main images and 5 µm for insets. Representative images are shown from the TRIS + Veh group to illustrate general morphology independent of amyloid injection.

**Supplementary Table 1. Correlation between cholinergic cell loss and glia markers**

|  | | **3-day** | | **12-day** | | **33-day** | |
| --- | --- | --- | --- | --- | --- | --- | --- |
|  |  | r | p | r | p | r | p |
| **IBA1**  **NBM** | Area occupied | -0.386 | 0.155 | -0.120 | 0.659 | -0.338 | 0.309 |
|  | Number of cells | +0.095 | 0.737 | -0.377 | 0.150 | -0.335 | 0.313 |
|  | Perimeter | +0.286 | 0.302 | -0.363 | 0.167 | -0.260 | 0.441 |
|  | Number of branches | +0.884 | 0.000 | +0.356 | 0.176 | -0.132 | 0.699 |
|  | Number of endpoints | +0.698 | 0.004 | +0.125 | 0.645 | -0.201 | 0.554 |
| **GFAP NBM** | Area occupied | -0.655 | 0.008 | -0.115 | 0.671 | +0.643 | 0.033 |
|  | Number of cells | -0.683 | 0.005 | -0.053 | 0.846 | +0.725 | 0.012 |
|  | Perimeter | -0.655 | 0.008 | -0.036 | 0.894 | +0.741 | 0.009 |
|  | Number of branches | -0.625 | 0.013 | -0.012 | 0.966 | +0.698 | 0.017 |
|  | Number of endpoints | -0.642 | 0.010 | -0.015 | 0.957 | +0.717 | 0.013 |

Red letters: significant differences. Abbreviations: IBA1: Ionized calcium-binding adaptor molecule 1; GFAP: glial fibrillary acidic protein
